# Recent Advances and New Perspectives in Capillary Electrophoresis-Mass Spectrometry for Single Cell “Omics”

**DOI:** 10.3390/molecules24010042

**Published:** 2018-12-22

**Authors:** Kellen DeLaney, Christopher S. Sauer, Nhu Q. Vu, Lingjun Li

**Affiliations:** 1 Department of Chemistry, University of Wisconsin-Madison, 1101 University Avenue, Madison, WI 53706, USA; kdelaney3@wisc.edu (K.D.); csauer@wisc.edu (C.S.S.); nqvu@wisc.edu (N.Q.V.); 2 School of Pharmacy, University of Wisconsin-Madison, 777 Highland Avenue, Madison, WI 53705, USA

**Keywords:** mass spectrometry, single-cell analysis, capillary electrophoresis, microchips, proteomics, metabolomics

## Abstract

Accurate clinical therapeutics rely on understanding the metabolic responses of individual cells. However, the high level of heterogeneity between cells means that simply sampling from large populations of cells is not necessarily a reliable approximation of an individual cell’s response. As a result, there have been numerous developments in the field of single-cell analysis to address this lack of knowledge. Many of these developments have focused on the coupling of capillary electrophoresis (CE), a separation technique with low sample consumption and high resolving power, and mass spectrometry (MS), a sensitive detection method for interrogating all ions in a sample in a single analysis. In recent years, there have been many notable advancements at each step of the single-cell CE-MS analysis workflow, including sampling, manipulation, separation, and MS analysis. In each of these areas, the combined improvements in analytical instrumentation and achievements of numerous researchers have served to drive the field forward to new frontiers. Consequently, notable biological discoveries have been made possible by the implementation of these methods. Although there is still room in the field for numerous further advances, researchers have effectively minimized various limitations in detection of analytes, and it is expected that there will be many more developments in the near future.

## 1. Introduction

With the rapid advancements in biopharmaceutical research, the possibilities of modern medicine are drastically expanding. Along with the new developments in this area of research, a variety of new therapies are becoming possible, and those that previously required large time investments and precious resources are now more readily available. A natural consequence of these substantial developments is that the demand for deeper levels of understanding of biological mechanisms has intensified. Overall research trends show a push toward understanding cellular heterogeneity, or the endogenous biological differences between cells within a population, in order to treat diseases and biological disorders more effectively with targeted, personalized therapeutics [1]. Although heterogeneity can result from a variety of reasons—most often stochastic in nature such as differences in gene, transcript, or protein expression—it is important to characterize these differences because they can lead to differences in the way cells respond to various treatments [2]. As a result, researchers are continuously developing new methodologies to probe the biomolecular content of single cells and profile differences between neighboring individual cells. This form of analysis carries with it numerous challenges regarding sensitivity and overall robustness of the technology due to the unavoidable small sample size inherent to individual cells. Therefore, many efforts have been focused on addressing these issues in a variety of contexts in order to expand our ability to characterize these cells.

Certain methods of analysis lend themselves better to addressing challenges at the single-cell level. For example, the incorporation of polymerase chain reaction (PCR) has made single-cell transcriptomics a routinely performed experiment [3]. However, other areas of analysis, such as metabolomics and proteomics, still require sufficient amounts of sample in order to yield confident detection of the different biological molecules present. As a result, these areas have lagged behind transcriptomics and much of what relates to single-cell analyses remains a mystery. Still, every year, more developments are being made in order to characterize the proteome and metabolome of individual cells, and continuous analytical advancements in other biological areas are further expanding the limits of the sample types that researchers can characterize [4,5]. Table 1 summarizes some key challenges related to each section.

The most promising technique for addressing the high demands of making single-cell measurements and understanding cellular heterogeneity is mass spectrometry (MS), due to its ability to rapidly interrogate virtually all analytes in a sample in a single analysis, providing both quantitative and structural information. Consequently, much development in recent years has focused on adopting the technique for sensitive detection of low-abundance analytes in the presence of complex biological matrices [5,34]. A highly fruitful coupling scheme has been with capillary electrophoresis (CE), in which CE provides an online or offline separation mechanism. CE has been successful in single-cell analyses because it involves minimal sample consumption, typically on the order of several nanoliters, and offers highly-resolved separations with desirable peak shapes, often on very short time scales. This successful pairing has been noted by the scientific community and has undergone numerous advances over the last several years to push the capabilities of CE-MS to reach lower detection limits, higher-complexity samples, and improved quantitative capabilities [35,36].

**Figure 1:**
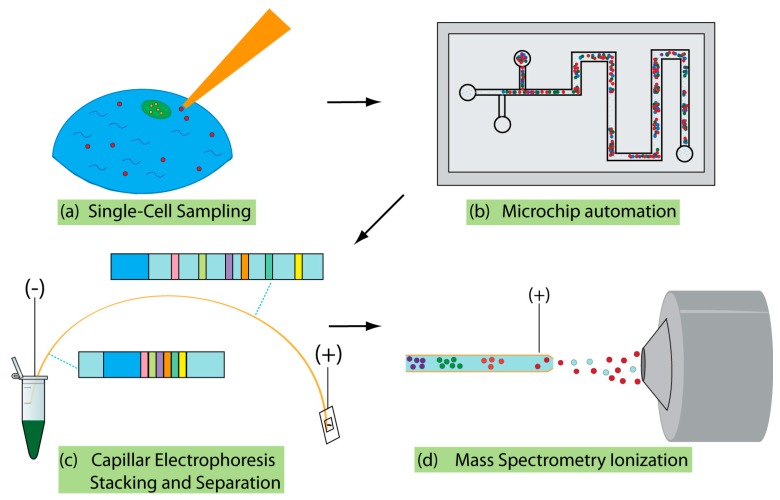
Diagram showing typical components of single-cell CE-MS analysis workflow, including (**a**) sampling, (**b**) microchip sample manipulation, (**c**) CE separation, and (**d**) ionization for MS acquisition. Not all single-cell workflows include all of these steps.

This review aims to provide a summary of recent developments in various aspects of single-cell CE-MS, as well as offer insight into the directions in which the field is advancing. The review is organized based on the typical components of a single-cell analysis workflow, which includes sections focusing on recent analytical advancements related to cell-sampling, automation, separation, MS acquisition, and data analysis. Additionally, we provide an overview of several novel applications using the coupling of CE-MS to provide context to the types of biological questions that are now able to be answered with advancements in the technology. The various sections are depicted in Figure 1, which outlines the overall coverage of the review. As there have been many noteworthy developments in recent years, we do not intend for this review to be a comprehensive report on all recent publications related to the area. Instead, this review should serve as an overview highlighting key advancements that are likely to shape the future of single-cell analysis in the upcoming years.

## 2. Addressing the Challenges of Single-Cell Sampling

A key challenge with performing analyses at the single-cell level is delivering the small volume of a cell to the instrument without introducing excessive sample loss. As cells typically contain only several picoliters or less of volume, it becomes difficult to distinguish between what is absent from a cell’s content and what is merely below the limit of detection. Due to the limited volume, the process of sample handling becomes especially important to ensure that maximum cell content is being retained up to the MS analysis step. As such, there have been numerous developments in recent years toward improvements in sampling and extraction efficiency prior to separation and analysis.

There are three key methods to acquire sample for single-cell analysis, including direct analysis, single-cell dissociation/culture, and targeting of specific single cells. Direct analysis through MS imaging—where the tissue section of interest is affixed to a surface and cells are directly interrogated with an ionization technique—is useful for retaining spatial context of the analyzed cells and minimizing sample preparation. Though the method lends itself to rapid, straightforward analysis, it is difficult to couple the method with separation techniques, as any separation would have to be done post-ionization. As such, much progress in the area of direct tissue analysis has incorporated gas-phase separation methods such as ion mobility separation [37]. However, there have been recent advancements in MS imaging instrumentation that lend themselves to other separation mechanisms [38] and it is likely only a matter of time before MS imaging and CE become readily coupled.

Even with current instrumentation, developments have been made in combining the advantages of direct tissue analysis with the separation power offered by CE. A notable example is the coupling of matrix-assisted laser desorption/ionization (MALDI) with CE-electrospray ionization (ESI) developed by Comi et al., in which MALDI is used to rapidly interrogate cells, and then liquid microjunction extraction is used to extract cells of interest for quantitative CE-ESI-MS [8]. The method was applied to single pancreatic islet cells and proved itself successful in classifying individual cells as either type alpha or beta prior to more thorough analysis by CE-MS [8]. The method shows a promising future in combining the benefits of two powerful ionization methods to parse out maximum information at the single-cell level. Another development by the same research group used an optically-guided MALDI-MS method to improve throughput of profiling of single cells [9]. Do et al. were able to rapidly perform classification of cell types, resulting in observed heterogeneity in the detected peptides and proteins [9]. Although this method did not involve CE separation, the integration of CE into the workflow could provide improved classification of the cell subpopulations.

**Figure 2:**
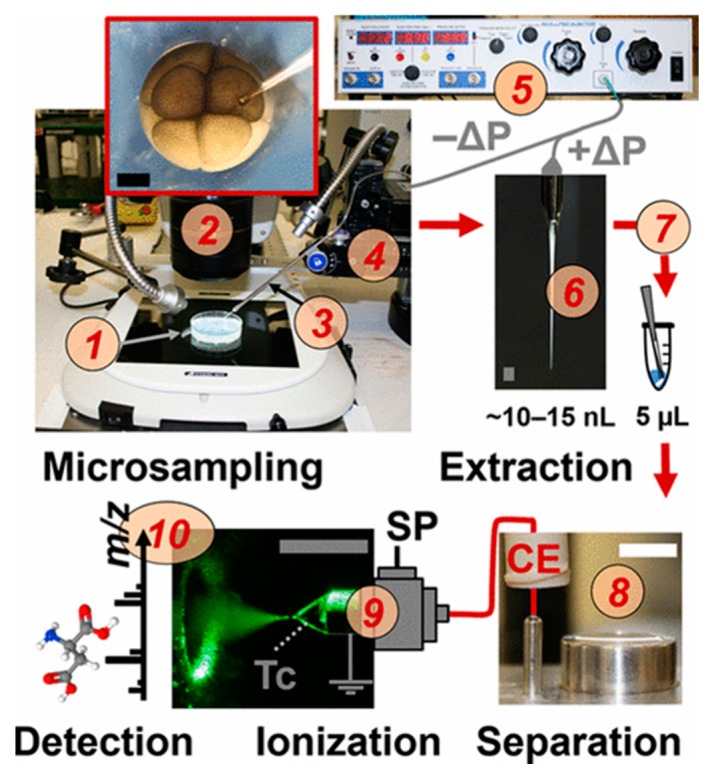
Depiction of in situ single-cell CE-MS with microprobe extraction, including sampling from individual frog embryonic cells, extracting of metabolites at the microscale, and online CE separation coupled to electrospray ionization (ESI)-MS for analysis of differentiating cells in live embryo. The live embryos (1) were identified using a stereomicroscope (2) and 10–15 nL portions were aspirated into a capillary (3) controlled by a multi-axis translation stage (4) coupled to a microinjector that supplied a vacuum (5). Afterwards, the collected cells in the capillary (6) were deposited into a vial via pressure-injection for metabolite extraction (7). After extraction, metabolites were measured with a microloading CE platform (8) and ionized for MS with a CE-ESI source (9). High-resolution tandem MS was used to identify and quantify metabolites (10). Reprinted with permission [7]. Copyright 2018 American Chemical Society.

Progress has also been made in utilizing the principles of CE for controlled sampling of small volumes within a cell for subsequent analysis. Yin et al. successfully employed localized electroosmotic extraction to sample sub-picoliter volumes from live cells using two electrodes and a finely-pulled capillary [6]. With their method, they successively detected over 50 metabolites from 5 pL of sample from *Allium cepa* [6]. Another method, utilizing microprobe aspiration coupled to CE-MS, was also able to successfully sample from live cells, namely frog embryos [7]. By integrating microsampling, metabolite extraction, and CE-MS, Onjiko et al. effectively minimized chemical interference and ion suppression, resulting in the detection of 70 known metabolites [7]. Figure 2 shows a depiction of the setup used for the microprobe single-cell CE-MS setup. These methods indicate the potential of single-cell CE-MS to further push the limits of our understanding of cellular metastasis and heterogeneity.

Though not necessarily exclusive to CE analysis, there have been notable advancements recently in the manner in which single cells are sampled. The two common challenges addressed include efficiency, so as to not introduce excessive sample loss or consumption, and cell discrimination, which is to ensure that sampled cells belong to specific subpopulations, etc. Capillary microsampling with a 200 µm tip was used by Zhang et al. to probe individual neurons, enabling them to analyze peptides in the cytoplasm and nucleus of each neuron [10]. With this method, they analyzed approximately 1.5 pL from the cytoplasm and 0.4 pL from the nucleus, leading to the identification of six neuropeptides and one novel peptide.^15^ A novel probe for in vivo analysis of single cells was recently developed that incorporates solid-phase microextraction (SPME) coated on the surface of the probe [11]. The probe had a diameter of smaller than 5 µm, enabling it to enrich for lipids in living cells from a precise position prior to analysis with nanoESI-MS [11]. The advantage of a short extraction time (i.e., 60 s) is that it results in a shorter analysis time, thus greater throughput, and less sample consumption, while still maintaining comparable results to other methods involving direct infusion [11]. Another novel sampling probe, denoted “single-probe,” was also developed and recently applied to algal cells under various conditions. This probe consists of a dual-bore quartz tubing that was pulled and fused to a silica capillary for nanoESI-MS analysis [12,13]. The probe showed highly promising results for sampling metabolites and peptides from live single-cells, with over 500 metabolites and peptides detected [17,18]. Traditional capillary microsampling with ESI-IMS-MS was also coupled with fluorescence microscopy to discriminate and select hepatocellular carcinoma cells in specific stages of cell mitosis in order to profile the cellular heterogeneity of dividing cells [39]. By coupling the two methods, metabolites and lipids in single cell subpopulations were profiled and correlated to the different mitotic stages [39]. Another notable strategy to improve the efficiency of single-cell sampling has been through the use of ionic liquids for microscale extraction [40]. By using an optimized ionic liquid extraction/dispersion method with subsequent sonication, Jha et al. achieved detection limits of less than 1 µg/L with accurate quantitation [40]. The authors demonstrated the efficacy of this novel method in the analysis of 15 neurotransmitters in individual cell samples in less than 15 min of analysis time [40]. This method was demonstrated as coupled to liquid chromatography (LC) but coupling to CE separation can be envisioned in future applications. Each of the methods described above demonstrates promising results that could potentially be expanded by the incorporation of CE separation.

## 3. Microchip Single-Cell Analysis—Toward the Automation of Sample Handling

As the small volumes and limited sample amounts associated with single-cell analysis lend themselves to substantial challenges, much effort has been made toward automating the sample preparation and CE process. Numerous microfluidic lab-on-a-chip devices have been developed and incorporated into the single-cell analysis workflows, enabling rapid, sensitive analysis of cells and improving the overall throughput and reproducibility of analyses.

**Figure 3:**
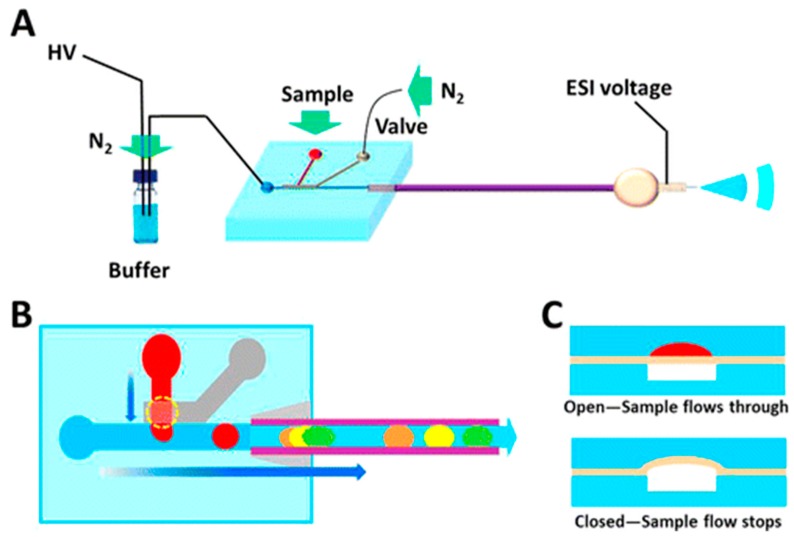
Schematic of hybrid microchip-CE setup for highly controlled, reproducible sample injections through the use of a hydrodynamic pneumatic-microvalve apparatus (**A**). Sample injection and electrophoretic separation was performed via switching of the valve (**B**). Channels of flow (red) and control (white) were separated by a membrane in both open and closed states (**C**). Reprinted with permission from https://pubs.acs.org/doi/abs/10.1021/ac501910p [14]. Adapted with permission from the American Chemical Society.

The clearest advantage to microchip automation is increasing sample throughput, which is important for the large populations necessary to be sampled in order to provide meaningful classifications and profiling of cell subtypes. Improvements in both speed and multiplexing have been demonstrated recently with a wall-less nano-electrophoretic device [17]. The device was constructed with open channels that avoid adsorption and allow the simultaneous electrophoretic separation of up to 100 individual cells in parallel channels, with the separation happening in as little as 10 ms [17]. Though the method only demonstrated proof-of-concept, it shows high potential toward greater depths of single-cell detection. Another device consisting of a hybrid polydimethylsiloxane (PDMS) microchip-CE setup utilizes a pressurized sample channel with a pneumatic microvalve that injects sample onto the capillary for capillary zone electrophoresis (CZE) separation [14]. This hybrid device demonstrated high throughput with reproducible and well-controlled injections [14]. Figure 3 shows a schematic of the device, in which the sample introduction step takes place on the PDMS microchip and the separation takes place in the seamlessly-attached capillary. In order to address inherent challenges from automated single-cell analysis, such as controlling the number of cells injected and reducing cell aggregation, a microchip that incorporated a double nano-electrode cell lysis was developed [16]. After the high-voltage cell lysis, the cell contents are subjected to electrophoretic separation and then ionized from the chip with a nanoESI emitter [16]. By automating the entire process and condensing it to a single chip, consecutive streamlined analyses are enabled [16].

The applicability of microchip automation was further expanded by incorporating single-cell labeling and derivatization into the process [19]. Peng et al. fabricated a chip comprised of double-helix microchannels that demonstrated an improved mixing efficiency, making the incorporation of additional reactive steps such as derivatization prior to electrophoretic separation possible without any additional pumping systems [19]. Quan et al. used an ionic liquid approach to incorporate labeling prior to CE separation [20]. By sandwiching the sample plug between leading and terminal ionic liquid plugs, analyte diffusion was effectively prevented prior to CE separation [20]. Another method utilized a pneumatic microvalve array, similar to the one described previously, which seamlessly incorporates all sampling handling steps [21]. With this method, mixing, transferring, and washing can be performed within a single run, with the results being similar to that of conventional CE sample preparation [21].

Another important advancement towards improving quantitation of single cells by microchip-CE analysis has been the development of consecutive gated injections. Li et al. utilized this novel injection method to enhance abilities in single cell manipulation in order to ensure constant-volume flow rates, thereby ensuring quantitative reproducibility [15]. With this method, the researchers reported consecutive absolute quantitation of superoxide anion and nitric oxide levels in single cells [15].

With the rapid expansion of single-cell microchip electrophoretic devices recently, Pan et al. performed a systematic study to characterize the relationship between microwell geometry and protein transport [41]. By comparing the time for diffusion and advection in each well, the researchers found that large cells, such as mammalian cells, are influenced by the geometry, and rectangular geometry, as opposed to the widely-used circular geometry, was shown to maximize separation performance in single-cell polyacrylamide gel electrophoresis (PAGE) [41]. It is clear that the field would greatly benefit from more systematic studies such as this to determine the effect of variables on the performances of microchip devices.

There have also been numerous advancements in microchip automation that do not necessarily incorporate CE, but either can seamlessly be incorporated with the technique or show great potential in the general field of single-cell ‘omics.’ One example is a chip device where a droplet containing a single cell is encapsulated in a well by an oil droplet, with an air pocket separating the oil and aqueous layers [22]. This setup miniaturizes the sample preparation stages by enabling all steps to be performed on a single surface, minimizing sample loss through adsorption, while also eliminating sample loss to the oil surface layer [22]. The oil cover gives protection against evaporation of the nanoliter droplet without interfering with subsequent separation and MS analysis [22]. The method currently has only been demonstrated with LC-MS, but replacing the LC separation with CE seems feasible. Another chip utilizes the dean flow phenomenon, in which a spiraled microchannel separates cells based on the drag force acting upon them as they move through the curvature, in order to sort cells while reducing agglomeration and uneven distributions, making quantitation more accurate [18]. Further optimization of the device is necessary for analyses other than lipidomics however, including the integration of a separation step prior to ESI. Other improvements include the incorporation of solid phase and monolithic-based micro-extraction directly on the chip, which has been particularly useful for the measurement of trace metals in single cells [42,43].

Overall, the recent advancements in microfluidic automation have drastically increased researchers’ abilities to manipulate single cells to gain deep molecular profiling of various analytes. Although numerous improvements have been made in sample handling, detection sensitivity, quantitative accuracy, and throughput, it is likely that these areas will only continue to improve exponentially in the near future.

## 4. Separations Prior to MS

Efficient, high-resolution separations are required for single-cell mass spectrometry to ensure reproducibility between highly heterogeneous samples and low volumes of analytes—typically less than 1 ng of protein [27]. Mass spectrometry offers high sensitivity, making it ideal for analyzing small sample sizes, but proteome complexity can often result in complicated spectra with many low-abundance analytes not being selected for further fragmentation and analysis by tandem MS (MS/MS). This leads to inconclusive data, low reproducibility between technical replicates, and ultimately low confidence in analyte identifications. As such, a suitable separation method is required to extend the analysis time and enable further depth of coverage of the single cell proteome, peptidome, metabolome, etc. Typically, LC and its variations (HPLC, UPLC, nanoLC) are the most commonly used separation methods for various -omic studies. LC is not ideal for volume limited samples, however, as it requires a greater injection volume (typically several microliters) and samples are heavily diluted by the mobile phase, lowering the effective concentration of the analyte as it reaches the mass spectrometer.

Electrophoretic separations, namely CE and microchip CE, offer unique advantages over chromatographic separations. These separation methods often require only a few nanoliters of volume, which is far more compatible with single-cell analyses. Additionally, separation methods have been developed to facilitate sample stacking and decrease band broadening to further enhance the already high resolution of CE separations and concentrate dilute samples without additional sample preparation. Further work is being done to reduce sample dilution and increase sensitivity. Due to their low sample volume requirements, high resolution, and high sensitivity, electrophoretic separations have quickly become the separation method-of-choice for single-cell analyses.

In recent years, there have been substantial improvements in the separation of small samples by electrophoresis. As single-cell analyses are sample limited, the samples often need to be analyzed in a single run, otherwise known as single-shot analysis. The Dovichi Lab has made many advancements in single-shot analyses using CE-MS and have produced results with higher sensitivity and resolution than nanoLC [23,24,25,26]. The group used linear polyacrylamide (LPA) as a neutral coating on the inner wall of the capillaries to both reduce electroosmotic flow (EOF) and analyte adsorption. Additionally, they lengthened the separation capillary from 40 to 60 cm. Reducing EOF and lengthening the capillary resulted in a longer separation, providing enhanced temporal resolution and acquisition of more MS spectra. Using these optimized parameters, the group was able to scale down the separation and MS analysis of *Escherichia coli* protein digests to 1 ng—approaching the protein levels of single cells. Performing triplicate analyses, they were able to identify 627 peptides from 142 protein groups, which they found to be comparable to, if not better, than nanoLC analyses (342 peptides from 140 protein groups) [23]. Their work was later expanded to human cell lines and HeLa cell lysates. These projects, however, used greater sample sizes (≈300 ng) to demonstrate the high depth of coverage achievable by single-shot high resolution MS [24,25]. Furthermore, Ludwig et al. demonstrated the technology’s use for analyzing the phosphoproteome, identifying over three times as many protein groups from 2 ng MCF-10A human mammary cell lysate digests as they did using UPLC [26]. The advances in single-shot analyses address many of the challenges presented by single-cell analyses; they have enhanced sensitivity, lowered injection volumes, and reduced sample processing steps to introduce sample loss. Though less common, fractionation prior to CE separation has been incorporated by some groups to improve proteome coverage. The Nemes group has demonstrated enhanced sensitivity when fractionating 1–20 µg neuronal protein digest with a reversed-phase column (ZipTip). Taking aliquots from individual fractions, they were able to identify 737 protein groups from 1 ng of digested protein and 225 protein groups from 500 pg, approximated to be a single neuron [27]. Their method displays high sensitivity and scalability for small samples, so although it is not currently reflective of a true single-cell analysis, the method is a major step towards this research goal.

Advancements in improving the lower limits of detection and reproducibility of electrophoretic separations has increased incorporation of CE into single-cell MS workflows, but efficient separation of low concentrations of biomolecules remains challenging. Various interesting methods have been developed to improve and adapt electrophoretic separations to make them better suited to measuring low-abundance analytes. For example, sample stacking provides many benefits for interfacing separations with MS. The analytes still have sufficient temporal resolution to allow more tandem MS spectra to be acquired, therefore increasing identifications while maintaining narrow peaks to minimize issues with co-migration of analytes. Sample stacking also allows more sample to be injected, so a greater number of ions reach the mass spectrometer, lowering the instrumental sensitivity required to accurately analyze them. Liu et al. devised a large volume sample stacking (LVSS) online concentration method in which they injected larger sample sizes onto the capillary and applied a high voltage across the separation capillary to pump out the excess buffer but left the sample on the capillary [28]. During LVSS, a large sample plug of dilute analytes is injected onto the capillary. The highly-conductive background electrolyte solution surrounding the plug forces the ions to move rapidly through the depletion zone in order to carry uniform current throughout the capillary. This large volume sample stacking method enabled the analysis of several endogenous nucleotides and metabolites and allowed the detection of analytes in only 51 fg of material. In their analysis of single *Xenopus laevis* embryo cells, the Nemes group employed field-amplified sample stacking (FASS) to greatly enhance the signal-to-noise ratio, resulting in a lower detection limit of 75 amol [29]. In FASS methods, samples are concentrated due to differences in conductivity between the background electrolyte buffer and the sample buffer. Samples are injected in a buffer of low conductivity and, therefore, high electric field. As the ions enter the comparatively low electric field of the background electrolyte buffer, their velocity decreases and they accumulate, increasing their local concentration. Another sample-stacking method used for small samples is the dynamic pH-junction. Here, a capillary is filled with an acidic background electrolyte and a large volume sample plug (in basic buffer) is hydrodynamically injected. The separation capillary has two pH boundaries—one at the injection end, and one at the interface between the sample plug and the initial background electrolyte. During the separation, the positively-charged hydrogen atoms at the injection vial begin to titrate the sample zone, causing the injection end pH boundary to move towards the other end of the capillary. The negatively-charged analytes in the sample plug move to the injection end and are focused at the pH boundary. When the two pH boundaries meet, the analytes are stacked into narrow bands, allowing for greater resolution despite the increased sample volume [30]. The Sun group has integrated dynamic pH junctions with both bottom-up and single-shot top-down proteomics [30,31]. The use of sample stacking enables a much greater sample volume to be injected, allowing for more identifications of proteins and proteoforms without sacrificing resolution.

**Figure 4:**
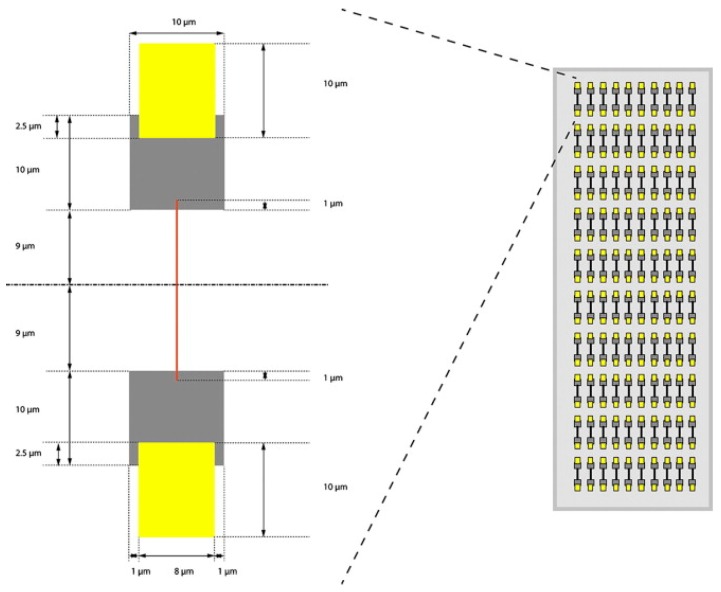
A schematic of the nano electrophoretic array device designed by Misevic et al. Paired nano wells joined by a 1 µm thick separation channel are arranged in a 10 × 10 array. Individual nano wells are then analyzed by secondary ionization mass spectrometry (SIMS) after a brief electrophoretic separation. Reprinted with permission [17].

In addition to capillary electrophoresis, there have been recent developments in chip-based electrophoresis in order to improve sensitivity and resolution of the separation. Misevic et al. have recently published a proof-of-concept paper, demonstrating two novel single-cell MS interfaces. The first is a nano electrophoretic array (NEA) that incorporates nL-sized wells that can efficiently separate cell contents in 10 ms. A schematic of the NEA is shown in Figure 4. The device consists of an array of paired nano wells, and the analytes are separated along a channel connecting the pairs of nano wells [17]. The second device the group has developed is a nano in micro array (NiMA) in which they use hydrophobic-coated nano wells to bind specific proteins of interest. The NiMA would not be suitable for discovery-based approaches but benefits from a higher sensitivity because unbound analytes are washed away. Both the NiMA and NEA employ secondary ionization mass spectrometry (SIMS) to provide high sensitivity and rapid analyses [17]. The devices have been tested only with protein standards, but the group has been able to use the devices to directly detect a single protein molecule, demonstrating a sensitivity that would make it highly applicable for single-cell analyses. Analysis of small samples (e.g., single-cells) requires the use of highly efficient separation methods with high resolution. Capillary electrophoresis and microchip CE have thus emerged as viable means to separate analytes of interest from the often complex background matrix prior to mass spectrometry analysis.

## 5. Interfacing CE with MS

Coupling CE to MS predominantly uses ESI for ionization, but the interface design itself varies greatly between labs. Typically, there are two types of interfaces, notated as sheath flow and sheathless interfaces. Sheath flow interfaces use a buffer liquid to establish the electrical connection necessary for the electrophoretic separation and electrospray ionization. The capillary outlet is placed in a metal emitter tip with buffer flowing around it to complete the circuit and provide enough solvent to form the Taylor-cone necessary for efficient ionization. Sheathless designs are similar but have a porous tip that allows for the transfer of solvent ions to complete the electrical connection, so no sheath buffer is required. Sheathless set-ups have no sample dilution caused by the presence of a sheath buffer, leading to enhanced sensitivity, but because there is minimal solvent emitted from the tip, the Taylor-cone is often unstable. This results in inconsistent ionization and poor reproducibility [44]. Recent efforts to make the electrospray more consistent in sheathless interface designs could enable their future use in single-cell analyses. The robustness of the sheath flow interface, however, has made this technique much more prevalent for single-cell and sample-limited analyses.

Many advances have been made in improving the CE-ESI interface to enhance sensitivity and improve analysis of small samples. Choi et al. recently developed a custom CE-ESI MS interface that utilizes a tapered-tip metal emitter. The geometry of this new design helps to better anchor the Taylor-cone, allowing for a much lower sheath flow rate—only 100–300 nL/min. This improves detection limits (as low as 260 zmol) by greatly reducing sample dilution and enhancing ionization. The tapered-tip, nanoflow design allowed the group to identify 1700 and quantify 800 proteins from 10 ng protein digest from a single cell of the *Xenopus laevis* embryo. Moreover, the results demonstrated high reproducibility and scalability to 1 ng protein digest [32]. The Nemes group has also utilized their custom-built CE-ESI interfaces for studying the metabolome of the *X. laevis* embryo. Using homemade microprobes to sample single cells, 10–15 nL of the cell content was hydrodynamically injected into the separation capillary. The electrophoretic separation was then coupled to high resolution MS with parallel reaction monitoring, providing a 60 amol lower limit of detection [29]. The same group later utilized the tapered-tip interface to examine the metabolome of differentiating cells in the *X. laevis* embryo. Due to non-normal tissue distributions often masking significant changes [45], multivariate statistical analyses, including PCA plots and ANOVA, were used to examine changes in metabolites between the 8-, 16-, and 32-cell embryos. The group was able to identify over 70 metabolites and determine statistically significant changes across cell growth stages [7].

**Figure 5:**
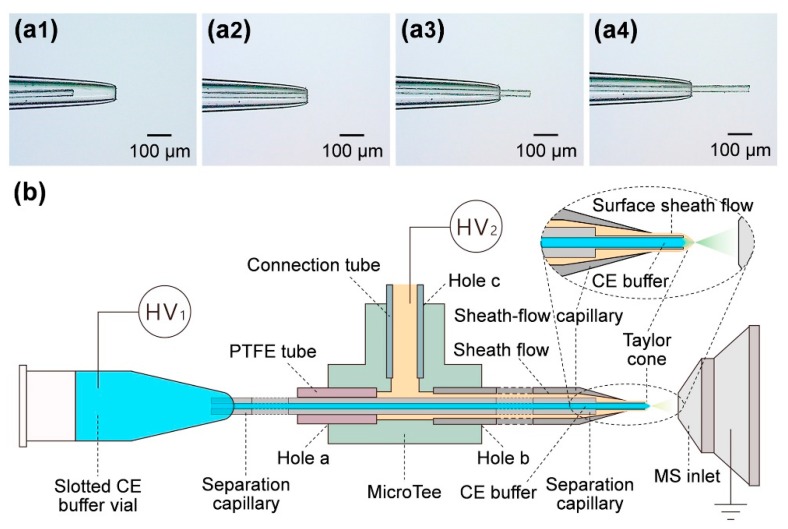
(**a1**–**4**) Multiple positions of the capillary tip were evaluated to optimize the interface. (**b**) A detailed schematic of the interface developed by Fang et al. Sheath buffer is supplied from a coaxial capillary and flows into the emitter tip surrounding the separation capillary. The sheath buffer flows around the outlet of the separation capillary that is extended past the emitter tip to generate the Taylor cone in the presence of an electric field. This ensures efficient and steady ionization of analytes with minimal dead volume. Reprinted with permission [33]. Copyright 2018 American Chemical Society.

Other groups have been working to improve the sheath flow design by minimizing the dead volume between the separation capillary and the emitter tip. At this junction, both dilution and diffusion of analytes occurs, lowering sensitivity. The Dovichi group has found that by etching the exterior of the separation capillary, they can place the outlet closer to the emitter tip orifice to improve sensitivity [25]. Fang et al. used a similar approach by testing a design where the separation capillary is extended from the emitter tip. There is enough room for the sheath flow to still supply a constant flow of solvent for the Taylor-cone, but there is less dead volume for dilution to occur, as shown in Figure 5. They estimate the dead volume to be as low as 4 pL (compared to 30 pL for prior designs) [33]. Though it has had minimal applications thus far, the high reproducibility, separation efficiency, and sensitivity of Fang’s design would make it ideal for future use in single-cell analyses.

Sheath flow CE-ESI is the most common interface, but other groups have developed interesting alternatives. Comi et al. have even integrated MALDI MS with CE-ESI MS to provide higher throughput analyses while still maintaining the quantitative abilities of CE-ESI. As described in a previous section, the group isolated pancreatic islets and deposited them onto indium-titanium oxide (ITO)-coated glass slides. Optical microscopy was combined with MALDI to analyze single cells, and based on the spectra acquired, each cell was determined to be an α or β cell [8]. Due to matrix interference, MALDI is not always suitable for analyzing metabolites and peptides with low molecular weights. As a result, the group extracted cellular contents using a custom-built liquid microjunction probe. The extraction was dried down to concentrate it, resuspended in a small volume (1 µL), and analyzed by CE-ESI-MS. This work allowed the group to isolate specific cell types and then compare the metabolic profile, signaling molecules, and protein precursors between the different cell types [8]. The novel interface was used for pancreatic islets but is suitable for many single cell analyses. Aside from using MALDI and ESI, SIMS has also been incorporated for sample-limited analyses. When evaluating their nanowell designs, Misevic et al. employed SIMS to provide enhanced ionization and sensitivity. They demonstrated the ability to detect a single protein molecule. Combining this impressive sensitivity with the rapid analysis that SIMS can provide, their interface would allow for many single-cell samples to be quickly separated by electrophoresis and then analyzed by MS, providing a high-throughput interface that could be used to analyze far more cells than previous designs.

Advances in sampling, separation, and MS analysis have enabled much growth in collecting data for single cell analyses. Interpreting the data of heterogeneous cell populations, however, is difficult and much less straightforward than looking at the average ensemble of the cells in a given sample. In conventional experiments looking at the average extracted analytes from a sample, Student’s *t*-tests and other univariate analyses are useful for determining significant differences. When trying to discern minute differences between cells, however, researchers have achieved better results employing principal component analysis, ANOVA, clustering analysis, and other multivariate statistical methods. The Nemes group has reported on the heterogeneity of the *X. laevis* embryo, and by using principal component analysis and clustering analysis, they have shown it is possible to differentiate metabolite patterns between different cells of the same embryo [7]. In another study, the Nemes group demonstrated the use of multivariate analysis to detect metabolic pathway differentiation between the left and right D1 blastomeres of the *Xenopus* embryo [46]. As data sets become larger, important patterns in heterogeneous cell populations become indistinguishable to the human eye. Recently, Liu et al. has integrated machine learning algorithms to enhance single-cell analyses. The group developed and trained an artificial neural network algorithm to predict drug-resistant cancer cell phenotypes [47]. The use of machine learning algorithms is an important step towards being able to accurately predict disease states for point-of-care treatments. These algorithms are much more effective than researchers’ judgement when it comes to discerning patterns in metabolic expression, allowing a suite of metabolites to be used to accurately predict phenotypes, rather than relying on specific biomarkers. By assessing multiple dependent factors, Liu’s algorithm demonstrated an impressive 87.1% predictive accuracy in characterizing drug-resistance in cancer cells [47]. Through machine learning and multivariate analyses, researchers are further improving analytical capabilities in single-cell analyses.

## 6. Novel Applications of Single-Cell CE-MS

The increased resolving capabilities of capillary electrophoresis have been utilized for interesting biological applications at the single-cell level. These advancements in CE have expanded our knowledge of metabolites, proteins, nucleotides, and unicellular organisms. The biological community is interested in studying the dynamics of these molecules within single cells in order to better define biological variability and individual sensitivity to disease. In the following sections, several interesting studies will be highlighted that showcase cellular heterogeneity at the single-cell level from the perspective of each biological sample type.

### 6.1. Metabolites

**Figure 6:**
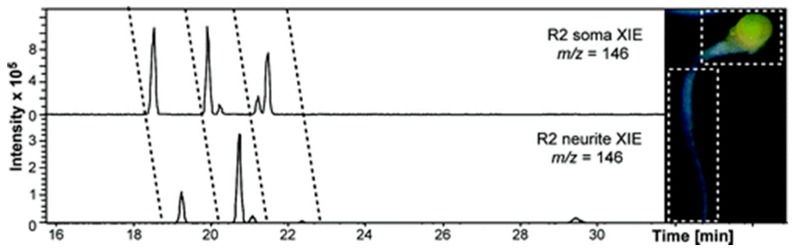
Different subcellular regions of the R2 neuron (neurite versus soma) yield different metabolite profiles. In this case, compounds with *m*/*z* 146 ± 0.5 Da are compared in the extracted ion electropherograms shown. Subcellular regions contain different relative amounts of several metabolites. Inset: Image of an isolated *Aplysia californica* R2 neuron and neurite. Reprinted with permission [48]. Copyright 2018 American Chemical Society.

**Figure 7:**
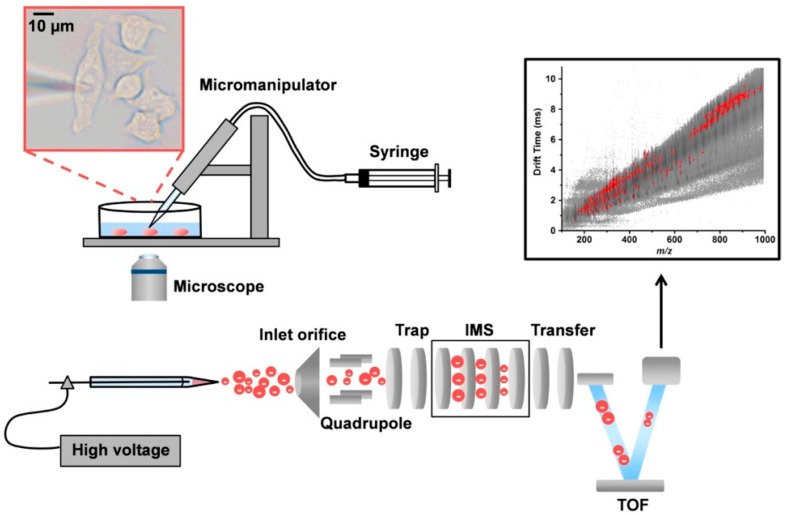
Schematic representation of experimental setup for single cell analysis using capillary microsampling ESI-ion mobility spectrometry (IMS)-MS. The pulled capillary is held by a micromanipulator and inserted into the cell. The cell content is then extracted with a syringe connected to the capillary. The inset shows a microscope image taken from an inverted microscope. After extraction, the capillary is filled electrospray solution, placed near the MS inlet, and subjected to a voltage to generate an electrospray. The resulting ions are separated by drift time (DT) ion mobility spectrometry (IMS) prior to MS analysis on a time of flight (TOF) MS. The resulting analytes are plotted by DT versus mass-to-charge ratio (*m/z*). Reprinted with permission from https://pubs.acs.org/doi/abs/10.1021/acs.analchem.5b02502 [52]. Further permissions related to the material excerpted should be directed to the American Chemical Society.

The composition and quantity of metabolites can be indicative of how well an individual is functioning. CE has commonly been coupled with mass spectrometry, gas chromatography (GC), and liquid chromatography in order to characterize metabolites in a variety of biological samples. For example, the high resolution capabilities of CE-MS allowed for the detection of novel metabolites within a wide *m*/*z* range, which was applied to better understand *Bacillus subtilis* sporulation [48]. The robustness and sensitivity of CE-MS was further developed when scientists were able to accurately measure metabolites from a specific organelle [49]. To achieve single-cell detection, the lowest limit of detection of CE-MS had to be reached. The advent of single cell analysis by CE-MS began when detection of biomolecules from large cells, such as neurons and embryos, was made possible. One of the earliest cases where this was achieved was by the Sweedler group, where they modified the sheath flow to control the flow rate of a CE-ESI-MS interface and lowered the sample volume required in order to make a reliable measurement, which enabled detection of metabolites (i.e., serotonin and acetylcholine) in single *Aplysia californica* neurons (Figure 6) [50]. Furthermore, CE-ESI-MS was able to detect the metabolomic differences between biochemically similar buccal B1 and B2 neurons [51]. Interestingly, they observed that freshly isolated neurons had cell type-dependent metabolites, yet cultured neurons did not follow this trend due to the microenvironment of the cell culture [51]. Another feat in CE-MS was achieved when the metabolic state of single human hepatocyte cells was able to be characterized by combining capillary microsampling and ESI-ion mobility separation (IMS)-MS, as depicted in Figure 7 [52]. This study involving a few hepatocyte cells revealed that insecticide (e.g., rotenone) leads to cell death through a cellular mechanism involving a shift in ATP levels [52].

**Figure 8:**
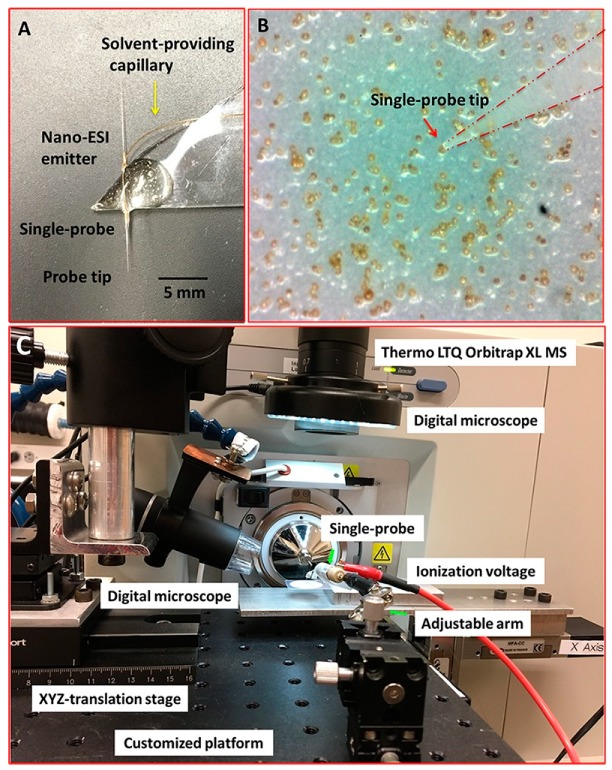
Experimental setup to measure single *Scrippsiella trochoidea* cells using the “Single probe” MS techniques. (**A**) Photograph of the Single-probe device with its different components labeled, including the probe tip and nano-ESI emitter; (**B**) image from microscope-linked camera used to target single *S. trochoidea* cell with the Single-probe; (**C**) setup used to manipulate the Single-probe MS device with components labeled. The Single-probe device was coupled to a Thermo LTQ Obitrap XL MS with an adjustable arm and digital microscope to control transfer of ions entering the MS. Reprinted with permission [12]. Copyright 2018 Sun, Yang and Wawrik.

Other ionization sources have been used in parallel with CE-MS to analyze single cells. Pancreatic islets were subjected to a novel workflow involving imaging analysis by MALDI-MS and subsequent liquid microextraction into the CE-ESI-MS system, from which novel metabolites (e.g., dopamine) were detected from single alpha and beta pancreatic cells [8]. Other groups have also developed platforms enabling automated detection of single cells. Specifically, a microfluidic chip was coupled with MS to characterize the fluctuation in expression of metabolites (e.g., dopamine and glutamic acid) in single PC-12 neuronal cells after the cells were exposed to stressors (e.g., KCl-induced polarization) [16]. The Nemes group has conducted a variety of studies that push the boundaries of CE-MS detection of single cells. Many of their recent studies focus on mapping the metabolome of a frog embryo as it develops from the 8-cell to the 32-cell stage while tracking the changes in expression of specific metabolites (i.e., acetylcholine, serine, arginine, and trolamine) [53]. Here, they discovered that daughter cells from a progenitor D1R cell had increased GABA and decreased aspartate levels [7]. They also observed differences in the metabolome between the left and right ventricle of the embryo in the 8-cell stage [54]. The success of the method developed by the Nemes group may launch a new subfield for studying the process of cell differentiation. Animal tissues are not the only sample types that have been analyzed by CE-ESI-MS. Individual phytoplankton cells were directly probed for metabolites prior to nano-ESI-MS analysis (Figure 8) [12]. Here, Sun et al. recorded the elemental differences between single algal cells that were subjected to stressors, such as exposure to illumination vs. darkness or cultured in replete vs. nitrogen-deficient conditions [12].

By using liquid microextraction coupled with ESI-MS, metabolites (GSH, AMP, ADP, ATP, UDP-Glc-NAc and GSSG) were detected and analyzed in single MCF-7 cells [55]. This was significant because they were able to simulate a disease state and measure the changes of these metabolites under a state of dysregulation, and they were able to track ATP, ADP, and AMP levels as a function of increasing cellular dysregulation [55]. In order to understand the physiological importance of the chirality state of amino acids with regard to their function as signaling molecules, the Sweedler group used LVSS-CE coupled with laser-induced fluorescence (LIF) to measure relative amounts of d-Glu and D-Asp in single *A. californica* neurons [56]. Here, they discovered that % d-Glu was lower than % D-Asp in all studied cells, and that d-Glu-containing neurons were found only in the F- and C-clusters of the cerebral ganglion [56]. Although they did not use MS detection, the sensitivity enhancement achieved by using LVSS described here can be developed for coupling with MS.

### 6.2. Proteins

Our knowledge of the proteome of single cells, such as neurons and blastomeres, has benefitted from the high resolution and sensitivity of CE. The first quantitative proteomics study in single blastomeres came from the Nemes group in which they identified and quantified 438 proteins groups from three different types of single frog blastomeres: D11, V11, and V21 [29]. Interestingly, most proteins from individual blastomeres of the same cell type (e.g., D11) overlapped, but the few cases where protein expression did not overlap potentially foreshadows the different fates of the daughter cells from the individual blastomeres [29]. Further, they were able to achieve coverage of approximately 30% of the predicted proteome for single *X. laevis* blastomeres [57]. Around the same time, others were also able to identify 808 protein groups from single blastomeres and followed the progression of these proteins as the embryo developed from the 2-cell stage to the 50-cell stage [58]. In this study, they saw an approximately two-fold decrease in protein groups detected between blastomeres from the 16-cell stage to the 50-cell stage due to the decrease in cell size, but they also observed greater protein abundance heterogeneity between cells at the 50-cell stage [58]. This large degree of heterogeneity is why identification of proteins from single mammalian neurons (~500 pg of proteins) is difficult. Recently, the detection capabilities of CE-MS were expanded in order to successfully identify 141 proteins from 500 pg of protein digest, and 737 proteins from 1 ng of protein digest [27]. To date, one of the smallest (approximately 20 µm) cell types to be analyzed by CE is PANC-1 cells, where researchers observed that phosphorylation rates of protein kinase B are slightly different for individual cells [59]. HeLa cells are a commonly used cell line and are similar in size to PANC-1 cells. A recent study used laser-induced fluorescence coupled to a capillary electroosmotic driven system to detect a human transmembrane protein (Her2) on a single HeLa cell [60]. The Herr group was able to analyze the proteins from the lysate of a single mammalian cell using single-cell polyacrylamide gel electrophoresis (scPAGE), a method that they improved by adding an electrophoretic injection of protein sample from a microwell at the head of the scPAGE separation axis and manipulating the microwell geometry [41]. This method can foreseeably be combined with MS as many proteomics workflows involve performing MS analysis of proteins directly from polyacrylamide gels. A novel modification of CE coupled with LIF detection involving a cell-permeable fluorescent activity-based probe (CpFABP)-labeling strategy allowed for the discovery of two different expression modes of cysteine cathepsin proteases among single RAW264.7 (leukemia) cells [61]. Additionally, by using CZE, the Allbritton group showed that glutaraldehyde fixation does not alter the phospholipid metabolism of single leukemic cells [62].

### 6.3. Other Types of Analytes

Due to the large size of *Aplysia* neurons (~1 mm), they have continued to be the model organism for other single cell CE studies. The low limit of detection of CE-MS allowed quantitation of 15 nucleotides and their derivatives from single *A. californica* neurons [28]. The limit of detection of CE-MS for nucleotides was pushed to its lowest limit (5 nM) by the Berezovski group when they were able to measure miRNAs from human serum [63].

Many other groups have made strides to improve analyte identification capabilities using CE-MS. The Dovichi group improved the number of peptide and protein identifications through CZE-ESI-MS/MS by 2.7-fold more than the previous record for single-shot analysis [64]. The previous record was 10,274 peptide IDs from 440 ng of HeLa digest, and the Dovichi group was able to generate over 27,000 peptide IDs from 220 ng of human K562 protein digest [64]. Using reverse phase (RP)-HPLC fractionation and sequential injections into a CE-MS system, the Lindner group was able to identify 62,000 peptides and more than 6100 proteins within 12.5 h, which is the highest number of peptides obtained for the analysis of a single proteome using CE-MS to date [65]. The Nemes group utilized CE to detect 217 nonredundant proteins from only 1 ng of protein digest from mouse cortex, in which key proteins involved in neurodegenerative disorders (e.g., parkinsonism and spastic paraplegia) were detected [32]. By using chip-based monolithic microextraction-ICP-MS, the amount of bismuth in a single HepG2 cell was calculated to be 0.62 pg/cell [43]. Additionally, by combining an inkjet sampling technique with CZE, individual HepG2 cells can now be separated with precise spatial and temporal control [66]. This method can be used to quantify single HepG2 cells with a more sensitive detector than UV detection, such as MS.

Beyond multicellular organisms, *Dictyostelium discoideum* are unicellular organisms that are similar in size (~2 mm) to *Aplysia* neurons. Recently, responses from oxidative stress from individual *D. discoideum* were measured using microfluidic chemical cytometry, in which they characterized the range of responses to hydrogen peroxide that individual cells experienced [67].

Recently, the preferential binding and binding sites of anticancer drugs NKP-1339, RAPTA-C and RM175 to either DNA or protein at single-amino acid and single-nucleotide resolution was characterized by CZE-ESI-MS [68]. Here, they saw that the anticancer drugs showed clear interactions with oligonucleotides and conditional binding with ubiquitin [68]. To the best of our knowledge, this is the only recent publication that explores multi-omic studies using CE-MS, but we anticipate more applications in this area in the near future.

## 7. Conclusions and Future Perspectives

As indicated in this review, single-cell analyses have benefitted greatly from the incorporation of CE and MS into experimental workflows. As such, much emphasis has been directed toward the development of various aspects centered around these two techniques, either independently or coupled to one another. From the beginning steps of a single-cell analysis, where individual cells’ contents must be acquired, developments have been made to improve the transmission of analytes and reduce sample loss. Microchip automation has further enhanced sample handling in order to introduce samples with greater precision and sensitivity into analytical instrumentation. Numerous research groups have greatly enhanced CE’s already remarkable resolving power to probe deeper into these limited amounts of sample, and novel MS interfaces have yielded greater ion transmission to maximize the information gained from each sample. These advancements have dramatically enhanced our biological understanding of individual cells, as demonstrated by improved knowledge of metabolites, proteins, nucleotides, etc., within cell subtypes. While sensitivity and sample complexity are still the greatest limitations to single-cell analysis, the combined effects of advanced analytical instrumentation and substantial achievements of researchers have effectively minimized these limitations. We expect to see these limitations become even smaller in upcoming years as researchers continue to make notable progress. One example of such progress is further development with the coupling of CE and MALDI, an area that has, as of yet, been under-explored but recently showed exciting promise in detection capabilities [69]. Coupling CE with MALDI MS imaging has enhanced the separation resolution to be comparable to online CE-ESI MS, further expanding the potential of this coupling for single-cell analysis [70,71]. Progress in this area has also demonstrated capabilities in the quantitation with the use of chemical tags [72]. With that, we also expect to see the technologies described herein further applied to address questions of cell heterogeneity and classify cell subtypes more confidently and accurately. Along with advancements in molecular profiling of individual cells, clinical research and developments in biomedicine will also benefit greatly, thus enabling improved therapeutics for individuals suffering from a variety of diseases and disorders.

## Figures and Tables

**Table 1:** Summary of some of the key challenges of single-cell capillary electrophoresis (CE)-mass spectrometry (MS) analysis, organized by section, as well as some notable strategies to address each challenge, accompanied by selected references. This table is intended to provide a general summary and not a comprehensive account of all developments to address each challenge.

Category	Key Challenges	Strategies	References
Sampling	Small-volume handling	Localized electroosmotic extraction	[6]
		Microprobe aspiration	[7]
	Cell discrimination/selection	MALDI-MS for guided sampling prior to CE-ESI-MS	[8,9]
		Novel probes for capillary microsampling	[10,11,12,13]
Microchip automation	Small-volume precision	Microvalve or gated sample injection	[14,15]
		On-chip high-voltage cell lysis	[16]
	Increasing throughput, reducing sample loss	Novel device geometries	[17,18]
		Including sample preparation steps on device	[16,19,20,21,22]
Separation	Increasing resolution and sensitivity	Reduce EOF and lengthen capillary to provide longer separation	[23,24,25,26]
		Fractionation prior to separation	[27]
	Limited sample amounts	Sample stacking for analyte pre-concentration	[28,29,30,31]
		Analyte enrichment via NiMA device	[17]
MS Interface	Sample dilution	Tapered-tip emitter enabling lower sheath flow rate	[32]
		Etched capillary allows for closer placement to the emitter orifice	[25,33]
	Increasing throughput	Optical microscopy and MALDI-MS cell screening	[8]
		Rapid separation with NEA microchip	[9]

## References

[1] Altschuler S.J., Wu L.F. (2010). Cellular heterogeneity: When do differences make a difference?. Cell.

[2] Smith S., Grima R. (2018). Single-cell variability in multicellular organisms. Nat. Commun..

[3] Shapiro E., Biezuner T., Linnarsson S. (2013). Single-cell sequencing-based technologies will revolutionize whole-organism science. Nat. Rev. Genet..

[4] Prakadan S.M., Shalek A.K., Weitz D.A. (2017). Scaling by shrinking: Empowering single-cell ‘omics’ with microfluidic devices. Nat. Rev. Genet..

[5] Zhang L., Vertes A. (2018). Single-Cell Mass Spectrometry Approaches to Explore Cellular Heterogeneity. Angew. Chem. Int. Ed. Engl..

[6] Yin R., Prabhakaran V., Laskin J. (2018). Quantitative Extraction and Mass Spectrometry Analysis at a Single-Cell Level. Anal. Chem..

[7] Onjiko R.M., Portero E.P., Moody S.A., Nemes P. (2017). In Situ Microprobe Single-Cell Capillary Electrophoresis Mass Spectrometry: Metabolic Reorganization in Single Differentiating Cells in the Live Vertebrate (*Xenopus laevis*) Embryo. Anal. Chem..

[8] Comi T.J., Makurath M.A., Philip M.C., Rubakhin S.S., Sweedler J.V. (2017). MALDI MS Guided Liquid Microjunction Extraction for Capillary Electrophoresis–Electrospray Ionization MS Analysis of Single Pancreatic Islet Cells. Anal. Chem..

[9] Do T.D., Ellis J.F., Neumann E.K., Comi T.J., Tillmaand E.G., Lenhart A.E., Rubakhin S.S., Sweedler J.V. (2018). Optically Guided Single Cell Mass Spectrometry of Rat Dorsal Root Ganglia to Profile Lipids, Peptides and Proteins. Chemphyschem.

[10] Zhang L., Khattar N., Kemenes I., Kemenes G., Zrinyi Z., Pirger Z., Vertes A. (2018). Subcellular Peptide Localization in Single Identified Neurons by Capillary Microsampling Mass Spectrometry. Sci. Rep..

[11] Deng J., Li W., Yang Q., Liu Y., Fang L., Guo Y., Guo P., Lin L., Yang Y., Luan T. (2018). Biocompatible Surface-Coated Probe for in Vivo, in Situ, and Microscale Lipidomics of Small Biological Organisms and Cells Using Mass Spectrometry. Anal. Chem..

[12] Sun M., Yang Z., Wawrik B. (2018). Metabolomic Fingerprints of Individual Algal Cells Using the Single-Probe Mass Spectrometry Technique. Front. Plant Sci..

[13] Pan N., Rao W., Kothapalli N.R., Liu R., Burgett A.W., Yang Z. (2014). The single-probe: A miniaturized multifunctional device for single cell mass spectrometry analysis. Anal. Chem..

[14] Kelly R.T., Wang C., Rausch S.J., Lee C.S., Tang K. (2014). Pneumatic Microvalve-Based Hydrodynamic Sample Injection for High-Throughput, Quantitative Zone Electrophoresis in Capillaries. Anal. Chem..

[15] Li L., Li Q., Chen P., Li Z., Chen Z., Tang B. (2016). Consecutive Gated Injection-Based Microchip Electrophoresis for Simultaneous Quantitation of Superoxide Anion and Nitric Oxide in Single PC-12 Cells. Anal. Chem..

[16] Li X., Zhao S., Hu H., Liu Y.M. (2016). A microchip electrophoresis-mass spectrometric platform with double cell lysis nano-electrodes for automated single cell analysis. J. Chromatogr. A.

[17] Misevic G.N., BenAssayag G., Rasser B., Sales P., Simic-Krstic J., Misevic N.J., Popescu O. (2014). Design and construction of wall-less nano-electrophoretic and nano in micro array high throughput devices for single cell ‘omics’ single molecule detection analyses. J. Mol. Struct..

[18] Huang Q., Mao S., Khan M., Zhou L., Lin J.-M. (2018). Dean flow assisted cell ordering system for lipid profiling in single-cells using mass spectrometry. Chem. Commun..

[19] Peng X., Zhao L., Guo J., Yang S., Ding H., Wang X., Pu Q. (2015). Double-helix micro-channels on microfluidic chips for enhanced continuous on-chip derivatization followed by electrophoretic separation. Biosens. Bioelectron..

[20] Quan H.H., Li M., Huang Y., Hahn J.H. (2016). A hydrophobic ionic liquid compartmentalized sampling/labeling and its separation techniques in polydimethylsiloxane microchip capillary electrophoresis. Electrophoresis.

[21] Jang L.-W., Razu M.E., Jensen E.C., Jiao H., Kim J. (2016). A fully automated microfluidic micellar electrokinetic chromatography analyzer for organic compound detection. Lab Chip.

[22] Li Z.Y., Huang M., Wang X.K., Zhu Y., Li J.S., Wong C.C.L., Fang Q. (2018). Nanoliter-Scale Oil-Air-Droplet Chip-Based Single Cell Proteomic Analysis. Anal. Chem..

[23] Zhu G., Sun L., Yan X., Dovichi N.J. (2013). Single-shot proteomics using capillary zone electrophoresis-electrospray ionization-tandem mass spectrometry with production of more than 1250 Escherichia coli peptide identifications in a 50 min separation. Anal. Chem..

[24] Sun L., Hebert A.S., Yan X., Zhao Y., Westphall M.S., Rush M., Zhu G., Champion M., Coon J., Dovichi N. (2014). Over 10000 Peptide Identifications from the HeLa Proteome by Using Single-Shot Capillary Zone Electrophoresis Combined with Tandem Mass Spectrometry. Angew. Chem. Int. Ed..

[25] Sun L., Zhu G., Mou S., Zhao Y., Champion M.M., Dovichi N.J. (2014). Capillary zone electrophoresis-electrospray ionization-tandem mass spectrometry for quantitative parallel reaction monitoring of peptide abundance and single-shot proteomic analysis of a human cell line. J. Chromatogr. A.

[26] Ludwig K.R., Sun L., Zhu G., Dovichi N.J., Hummon A.B. (2015). Over 2300 phosphorylated peptide identifications with single-shot capillary zone electrophoresis-tandem mass spectrometry in a 100 min separation. Anal. Chem..

[27] Choi S.B., Lombard-Banek C., Munoz L.P., Manzini M.C., Nemes P. (2018). Enhanced Peptide Detection Toward Single-Neuron Proteomics by Reversed-Phase Fractionation Capillary Electrophoresis Mass Spectrometry. J. Am. Soc. Mass Spectrom..

[28] Liu J.X., Aerts J.T., Rubakhin S.S., Zhang X.X., Sweedler J.V. (2014). Analysis of endogenous nucleotides by single cell capillary electrophoresis-mass spectrometry. Analyst.

[29] Lombard-Banek C., Reddy S., Moody S.A., Nemes P. (2016). Label-free Quantification of Proteins in Single Embryonic Cells with Neural Fate in the Cleavage-Stage Frog (*Xenopus laevis*) Embryo using Capillary Electrophoresis Electrospray Ionization High-Resolution Mass Spectrometry (CE-ESI-HRMS). Mol. Cell. Proteom..

[30] Lubeckyj R.A., McCool E.N., Shen X., Kou Q., Liu X., Sun L. (2017). Single-Shot Top-Down Proteomics with Capillary Zone Electrophoresis-Electrospray Ionization-Tandem Mass Spectrometry for Identification of Nearly 600 Escherichia coli Proteoforms. Anal. Chem..

[31] Yang Z., Shen X., Chen D., Sun L. (2018). Microscale Reversed-Phase Liquid Chromatography/Capillary Zone Electrophoresis-Tandem Mass Spectrometry for Deep and Highly Sensitive Bottom-Up Proteomics: Identification of 7500 Proteins with Five Micrograms of an MCF7 Proteome Digest. Anal. Chem..

[32] Choi S.B., Zamarbide M., Manzini C., Nemes P. (2017). Tapered-Tip Capillary Electrophoresis Nano-Electrospray Ionization Mass Spectrometry for Ultrasensitive Proteomics: The Mouse Cortex | SpringerLink. J. Am. Soc. Mass Spectrom..

[33] Fang P., Pan J.Z., Fang Q. (2018). A robust and extendable sheath flow interface with minimal dead volume for coupling CE with ESI-MS. Talanta.

[34] Comi T.J., Do T.D., Rubakhin S.S., Sweedler J.V. (2017). Categorizing Cells on the Basis of their Chemical Profiles: Progress in Single-Cell Mass Spectrometry. J. Am. Chem. Soc..

[35] Huang W.-H., Ai F., Wang Z.-L., Cheng J.-K. (2008). Recent advances in single-cell analysis using capillary electrophoresis and microfluidic devices. J. Chromatogr. B.

[36] Voeten R.L.C., Ventouri I.K., Haselberg R., Somsen G.W. (2018). Capillary Electrophoresis: Trends and Recent Advances. Anal. Chem..

[37] Sans M., Feider C.L., Eberlin L.S. (2018). Advances in mass spectrometry imaging coupled to ion mobility spectrometry for enhanced imaging of biological tissues. Curr. Opin. Chem. Biol..

[38] Buchberger A.R., DeLaney K., Johnson J., Li L. (2017). Mass Spectrometry Imaging: A Review of Emerging Advancements and Future Insights. Anal. Chem..

[39] Zhang L., Sevinsky C.J., Davis B.M., Vertes A. (2018). Single-Cell Mass Spectrometry of Subpopulations Selected by Fluorescence Microscopy. Anal. Chem..

[40] Jha R.R., Singh C., Pant A.B., Patel D.K. (2018). Ionic liquid based ultrasound assisted dispersive liquid-liquid micro-extraction for simultaneous determination of 15 neurotransmitters in rat brain, plasma and cell samples. Anal. Chim. Acta.

[41] Pan Q., Herr A.E. (2018). Geometry-induced injection dispersion in single-cell protein electrophoresis. Anal. Chim. Acta.

[42] Yu X., Chen B., He M., Wang H., Hu B. (2018). Chip-based magnetic solid phase microextraction coupled with ICP-MS for the determination of Cd and Se in HepG2 cells incubated with CdSe quantum dots. Talanta.

[43] Zhang J., Chen B., Wang H., Huang X., He M., Hu B. (2016). Chip-based monolithic microextraction combined with ICP-MS for the determination of bismuth in HepG2 cells. J. Anal. Atom. Spectrosc..

[44] Sanz-Nebot V., Balaguer E., Benavente F., Barbosa J. (2005). Comparison of sheathless and sheath-flow electrospray interfaces for the capillary electrophoresis-electrospray ionization-mass spectrometry analysis of peptides. Electrophoresis.

[45] Kharchenko P.V., Silberstein L., Scadden D.T. (2014). Bayesian approach to single-cell differential expression analysis. Nat. Method..

[46] Onjiko R.M., Morris S.E., Moody S.A., Nemes P. (2016). Single-cell mass spectrometry with multi-solvent extraction identifies metabolic differences between left and right blastomeres in the 8-cell frog (Xenopus) embryo. Analyst.

[47] Liu R., Zhang G., Yang Z. (2018). Towards rapid prediction of drug-resistant cancer cell phenotypes: Single cell mass spectrometry combined with machine learning. Chem. Commun..

[48] Soga T., Ohashi Y., Ueno Y., Naraoka H., Tomita M., Nishioka T. (2003). Quantitative metabolome analysis using capillary electrophoresis mass spectrometry. J. Proteom. Res..

[49] Chen Y., Arriaga E.A. (2007). Individual electrophoretic mobilities of liposomes and acidic organelles displaying pH gradients across their membranes. Langmuir.

[50] Lapainis T., Rubakhin S.S., Sweedler J.V. (2009). Capillary Electrophoresis with Electrospray Ionization Mass Spectrometric Detection for Single Cell Metabolomics. Anal. Chem..

[51] Nemes P., Knolhoff A.M., Rubakhin S.S., Sweedler J.V. (2012). Single-Cell Metabolomics: Changes in the Metabolome of Freshly Isolated and Cultured Neurons. ACS Chemical. Neurosci..

[52] Zhang L., Vertes A. (2015). Energy Charge, Redox State, and Metabolite Turnover in Single Human Hepatocytes Revealed by Capillary Microsampling Mass Spectrometry—PubMed—NCBI. Anal. Chem..

[53] Onjiko R.M., Portero E.P., Moody S.A., Nemes P. (2017). Microprobe Capillary Electrophoresis Mass Spectrometry for Single-cell Metabolomics in Live Frog (*Xenopus laevis*) Embryos. J. Vis. Exp..

[54] Onjiko R.M., Plotnick D.O., Moody S.A., Nemes P. (2017). Metabolic Comparison of Dorsal versus Ventral Cells Directly in the Live 8-cell Frog Embryo by Microprobe Single-cell CE-ESI-MS. Anal. Methods.

[55] Zhang X., Wei Z., Gong X., Si X., Zhao Y., Yang C., Zhang S., Zhang X. (2016). Integrated Droplet-Based Microextraction with ESI-MS for Removal of Matrix Interference in Single-Cell Analysis. Sci. Rep..

[56] Patel A.V., Kawai T., Wang L., Rubakhin S.S., Sweedler J.V. (2017). Chiral Measurement of Aspartate and Glutamate in Single Neurons by Large-Volume Sample Stacking Capillary Electrophoresis. Anal. Chem..

[57] Lombard-Banek C., Moody S.A., Nemes P. (2016). Single-Cell Mass Spectrometry for Discovery Proteomics: Quantifying Translational Cell Heterogeneity in the 16-Cell Frog (Xenopus) Embryo. Angew. Chem. Int. Ed. Engl..

[58] Sun L., Dubiak K.M., Peuchen E.H., Zhang Z., Zhu G., Huber P.W., Dovichi N.J. (2016). Single cell proteomics using frog (*Xenopus laevis*) blastomeres isolated from early stage embryos, which form a geometric progression in protein content. Anal. Chem..

[59] Mainz E.R., Wang Q., Lawrence D.S., Allbritton N.L. (2016). An Integrated Chemical Cytometry Method: Shining a Light on Akt Activity in Single Cells. Angew. Chem. Int. Ed. Engl..

[60] Geng X., Shi M., Ning H., Feng C., Guan Y. (2018). A compact and low-cost laser induced fluorescence detector with silicon based photodetector assembly for capillary flow systems. Talanta.

[61] Chen D., Fan F., Zhao X., Xu F., Chen P., Wang J., Ban L., Liu Z., Feng X., Zhang Y. (2016). Single Cell Chemical Proteomics with Membrane-Permeable Activity-Based Probe for Identification of Functional Proteins in Lysosome of Tumors. Anal. Chem..

[62] Proctor A., Sims C.E., Allbritton N.L. (2017). Chemical fixation to arrest phospholipid signaling for chemical cytometry. J. Chromatogr. A.

[63] Khan N., Mironov G., Berezovski M.V. (2016). Direct deposition of endogenous MicroRNAs and their post-transcriptional modifications in cancer serum by capillary electrophoresis-mass spectrometry. Anal. Bioanal. Chem..

[64] Zhang Z., Hebert A.S., Westphall M.S., Qu Y., Coon J.J., Dovichi N.J. (2018). Production of Over 27000 Peptide and Nearly 4400 Protein Identifications by Single-Shot Capillary-Zone Electrophoresis-Mass Spectrometry via Combination of a Very-Low-Electroosmosis Coated Capillary, a Third-Generation Electrokinetically-Pumped Sheath-Flow Nanospray Interface, an Orbitrap Fusion Lumos Tribrid Mass Spectrometer, and an Advanced-Peak-Determination Algorithm. Anal. Chem..

[65] Faserl K., Sarg B., Sola L., Lindner H.H. (2017). Enhancing Proteomic Throughput in Capillary Electrophoresis-Mass Spectrometry by Sequencial Sample Injection. Proteomics.

[66] Zhang W., Li N., Zeng H., Nakajima H., Lin J.M., Uchiyama K. (2017). Inkjet Printing Based Separation of Mammalian Cells by Capillary Electrophoresis. Anal. Chem..

[67] Rodogiannis K., Duong J.T., Kovarik M.L. (2018). Microfluidic single-cell analysis of oxidative stress in Dictyostelium discoideum. Analyst.

[68] Artner C., Holtkamp H.U., Hartinger C.G., Meier-Menches S.M. (2017). Characterizing activation mechanisms and binding preferences of ruthenium metallo-prodrugs by a competitive binding assay. J. Inorg. Biochem..

[69] Zhong X., Zhang Z., Jiang S., Li L. (2014). Recent advances in coupling capillary electrophoresis based separation techniques to ESI and MALDI MS. Electrophoresis.

[70] Wang J.H., Ye H., Zhang Z.C., Xiang F., Girdaukas G., Li L.J. (2011). Advancing Matrix-Assisted Laser Desorption/Ionization-Mass Spectrometric Imaging for Capillary Electrophoresis Analysis of Peptides. Anal. Chem..

[71] Zhang Z., Ye H., Wang J., Hui L., Li L. (2012). Pressure-assisted capillary electrophoresis coupling with matrix-assisted laser desorption/ionization-mass spectrometric imaging for quantitative analysis of complex peptide mixtures. Anal. Chem..

[72] Wang J., Zhang Y., Xiang F., Zhang Z., Li L. (2010). Combining Capillary Electrophoresis Matrix-Assisted Laser Desorption/Ionization Mass Spectrometry and Stable Isotopic Labeling Techniques for Comparative Crustacean Peptidomics. J. Chromatogr. A.

